# Ecofriendly silver nanoparticles biosynthesis from *Penicillium commune* NRC 2016-3 as antimicrobial agents for textile materials

**DOI:** 10.1038/s41598-025-10571-4

**Published:** 2025-07-15

**Authors:** Sayeda Abdelrazek Abdelhamid, Sahar S. Mohamed, Fatma Abdelghaffar

**Affiliations:** 1 Microbial Biotechnology Department, National Research Centre, Dokki, 12622, Giza, Egypt; 2 Dyeing, Printing, and Textile Auxiliaries Department, Textile Research and Technology Institute, National Research Centre, Dokki, 12622, Giza, Egypt

**Keywords:** Nano-silver, *Penicillium commune* NRC 2016-3, Antimicrobial, Fabrics, Biological techniques, Microbiology

## Abstract

The biological synthesis of silver nanoparticles (AgNPs) using fungi has received considerable attention, given that they are environmentally friendly, non-toxic, and inexpensive. Therefore, the current work investigates the biological synthesis of silver nanoparticles (AgNPs) using the extracellular filtrate of *Penicillium commune* NRC 2016–3. Physicochemical analyses confirmed the formation of AgNPs with a surface plasmon resonance peak at 420 nm. Transmission electron microscopy (TEM) and Scanning electron microscopy (SEM) revealed spherical particles ranging in size from 3.69 to 9.61 nm, with a zeta potential of -30.5 mV, indicating good stability. The biosynthesized AgNPs exhibited strong antibacterial and antifungal activity against various test microorganisms, with inhibition zones ranging from 30 to 33 mm for Gram-positive and 26 to 30 mm for Gram-negative bacteria. The minimum inhibitory concentration (MIC) and minimum bactericidal concentration (MBC) values were found to be 2–5 µg/mL and 20–30 µg/mL, respectively. Furthermore, the AgNPs-treated fabrics revealed good antimicrobial properties and demonstrated their durability even after multiple washing cycles. This finding highlights the potential of green-synthesized AgNPs for creating durable antimicrobial textiles.

## Introduction

Green nanotechnology, often referred to as “green nano,” is an environmentally friendly approach combining principles of green chemistry and green engineering. This method is used to study the synthesis and applications of nanomaterials in a sustainable way^1^. Green chemistry employs renewable resources such as plant extracts, fungi, and bacteria to produce non-toxic, eco-friendly nanoparticles (NPs) of metals like copper, palladium, zinc, silver, tin, gold, and titanium^2,3^. These nanoparticles, which range in size from 1 to 100 nanometers, have special physical features resulting from their small size, such as quantum confinement, high surface energy, and a huge surface area. These features make them highly effective in various applications while aligning with environmental sustainability goals^4^. Nanoparticles (NPs) are synthesized using stabilizing and reducing agents such as ketones, flavones, terpenoids, amides, and aldehydes, which are commonly found in the bioactive compounds of various organisms^1^. The source of these extracts plays a key role in shaping the distinct chemical, morphological, and physical properties of the nanoparticles produced^2^. Biosynthesized NPs offer several advantages over traditional methods, exhibiting enhanced antibacterial, antifungal, antiviral, anti-inflammatory, and anticancer properties. Additionally, they demonstrate superior photocatalytic degradation activity, making them highly effective in environmental and biomedical applications^3–5^. Previous studies indicated that environmentally friendly production is a remarkable methodology when compared with chemical production, delivering advantages such as the eco-friendliness of nanoparticles, large-scale synthesis, and the effectiveness of costs^1^. Among the noble metallic nanoparticles, silver nanoparticles (AgNPs) stand out because of their chemical inertness, biocompatibility, oxidation resistance, and strong antimicrobial properties^2,3^. AgNPs have widespread applications in biomedical, healthcare, food manufacturing, drugs, fabrics, and biosensors^6^. Fungi, such as *Penicillium citrinum*, *Penicillium chrysogenum*, and *Aspergillus fumigatus*, play a key role in the green synthesis of AgNPs. These fungi facilitate the production of highly effective, eco-friendly AgNPs, which have proven valuable in treating various medical conditions thanks to their potent antimicrobial activity^4,5^. As a result, AgNPs are now recognized as potent antimicrobial agents in numerous uses involving medical care, agriculture, and industry^7,8^. One of the most promising fields making significant use of nanotechnology presently is the textile industry^9,10^, where nanomaterials play a crucial role, particularly in textile finishing processes^11–13^. Overall, antibacterial textiles have piqued the interest of researchers in healthcare applications and medical clothing, particularly with the appearance of novel resistant microbe strains^14,15^. In the current study, various Egyptian microbial strains were evaluated to assess their potential for green biosynthesis of the AgNPs. Among the tested strains, the extracellular filtrate *P. commune* NRC 2016-3 proved to be the most effective in biosynthesizing AgNPs with strong antimicrobial properties. These biosynthesized AgNPs were then characterized and applied to different textile fabrics, demonstrating their potential to enhance antimicrobial textile applications.

## Materials and methods

### Chemicals

Silver nitrate (AgNO_3_) was purchased from Panreac (Spain), nutrient agar (NA) was from the Oxoid company, nutrient broth (NB) was from the Oxoid company, potato dextrose agar (PDA) was from the Biolap company, potato dextrose broth (PDB) was from the Biolap company, and the other chemical was from the Fluka company.

### Microorganisms

In the current study, we explore the biosynthesized silver nanoparticles using various microbe strains, including *P. commune* NRC 2016 strain, *Fusarium oxysporum* NRC 2017, *Bacillus velezensis* 3SME, and *Bacillus cereus* 3SME. These strains were previously isolated, identified, and deposited in the Gene Bank under accession numbers KU752217, MF62208, MW523035, and MW522550, respectively^16–18^. In our previous research, *P. commune* NRC 2016 and *F. oxysporum* NRC 2017 were exposed to mutagenic agents using gamma radiation (Ɣ ray), ethyl methane sulfonate (EMS), and ethidium bromide (Et Br), as indicated in Table 1^19^.

**Table 1:** The names of *P. commune* NRC 2016 and *F. oxysporum* NRC 2017 with various induced mutagenesis as our previous study^19^.

Name	Mutagen
*P. commune *NRC 2016	Wild type
*P. commune* NRC 2016-1	Ɣ ray
*P. commun*e NRC 2016-2	Et Br
*P. commune* NRC 2016-3	EMS
*F. oxysporum* NRC 2017	Wild type
*F. oxysporum* NRC 2017-1	Ɣ ray
*F. oxysporum* NRC 2017-2	Et Br
*F. oxysporum* NRC 2017-3	EMS

### Biosynthesis of AgNPs

The biosynthesis of AgNPs according to Othman et al.^20^. Fungal and bacterial isolates were grown in PDB and NB for 7 days at 30 °C and 2 days at 37 °C, respectively, at static conditions. Following that, the culture was filtered, and the resulting supernatant was applied to form the AgNPs synthesis. The extracellular filtrate of various microorganisms at a sufficient ratio with 1 mM AgNO_3_ (1:1) has been incubated at 30ºC, shaking, and dark factors for 24 h.

### Antimicrobial test of AgNPs using well diffusion assay

The antimicrobial features of the produced silver nanoparticles, the microbial filtrate (negative control), and the silver nitrate solution (control) have been examined against six test microorganisms using the Well diffusion assay^21^. The test microbes included *Bacillus subtilis* NRRL B-94, Staphylococcus *aureus* NRRL B-313, *Pseudomonas aeruginosa* NRC B-32, *Escherichia coli* NRC B-3703, *Aspergillus niger* NRRL 599, and *Candida albicans* NRRL477. In the current study, the test bacterial strains were cultivated on NA medium, while the fungal strains were cultivated on PDA. Bacterial strains underwent incubation at 37 °C for 24 h, and fungal strains at 30 °C for 48 h. A spreader was used to distribute the microbial inoculum equally across the agar plates; after that, a 6-mm-diameter well was formed by a sterile borer in the inoculation medium, and finally, a 10 µg/mL concentration of AgNPs was added. For two hours, the plates were refrigerated at 4 °C to allow the AgNPs to diffuse in an agar medium. The presence of inhibitory zones was determined, which indicated antimicrobial action. As reference antibiotics, ampicillin was employed against test bacteria and mycostatin against test fungi ((positive control). The data was statistically examined and provided as mean values ± standard errors.

### Determination of minimum inhibitory concentrations using the optical density assay

The highest antimicrobial activity was AgNPs biosynthesis using the extracellular filtrate of the *P. commune NRC* 2016-3 strain, so we completed our study on it. The test microbes included *S. aureus* NRRL B-313, *B. subtilis* NRRL B-94, *P. aeruginosa* NRC B-32, *E. coli* NRC B-3703, and C. *albicans* NRRL477. The MIC effect of the AgNPs was evaluated using the optical density assay for the different tested microbes by adding the AgNPs with concentrations of 1–5 µg/mL at 3 ml nutrient broth culture. The tubes were injected with 50 µL of each tested fresh microbial sample. The growth and sterility controls were two blank nutrient broth tubes with and without microbial inoculation, which were subsequently cultured in a shaking incubator at 150 rpm at 37 °C for 24 h for the bacterial strains and at 30 °C for 48 h for the candida isolate. Microbial growth was evaluated at 620 nm, and the results were presented as growth inhibition percentages. The first tube with no apparent development after the period of incubation was chosen as the MIC^22^.

### Determining minimum bactericidal and fungicidal concentrations

The minimum bactericidal concentration (MBC) and minimum fungicidal concentration (MFC) of AgNPs against the test microorganisms were determined using a method according to Cos et al.^23^. Each tube was inoculated with 50 µL of the adjusted fresh microbial strains. The AgNPs concentrations were 10–35 µg/mL in 3 mL nutrient broth culture. The MBC, or MFC, was defined as the lowest concentration of AgNPs that inhibited bacterial or fungal growth.

### Characterization of the prepared AgNPs with *P. commune* NRC 2016-3

Evaluation of physical, chemical, structural, and morphological features of the chemical AgNPs with *P. commune* NRC 2016-3 showed the highest antimicrobial properties. The physicochemical techniques include UV-Vis, TEM, SEM-EDX, XRD, FTIR, and zeta potential.

### UV-Visible spectra

The reduced amount of silver ions was visually observed for 24 h using *P. commune* NRC 2016-3 filtrate. To further analyze the optical properties of the biosynthesized AgNPs, the UV/VIS PC-Spectrophotometer (Shimadzu, UV-2401) covers the wavelength that ranges from 200 to 800 nm^24^.

### Transmission electron microscopy

The high-resolution transmission electron microscope (HRTEM, JEM-2100 F) from Japan has been utilized at 200 kV to confirm the creation of produced AgNPs with *P. commune* NRC 2016-3, in addition to their shape, size, and distribution^20^.

### Scanning electron microscopy

The surface morphology of the produced AgNPs with *P. commune* NRC 2016-3 was tested using scanning electron microscopy (SEM-EDX) (SEM FEG Quanta 250 Czech)^25^.

### X-ray diffraction

XRD was utilized to evaluate the structural properties of the produced AgNPs at room temperature (10 °C to 80 °C). An XRD equipment (X-Pert, PRO, and Panalytical Netherlands) was utilized to analyze the AgNPs produced via *P. commune* NRC 2016-3^25^.

### Fourier transforms infrared spectroscopy

The chemical structures of AgNPs produced *via P. commune* NRC 2016-3 have been determined. The Fourier transform for infrared-attenuated total reflection spectroscopy (FTIR-ATR) has been used extensively to study adsorption and surface reactions. The FTIR-ATR measurement was performed with a Bruker VERTEX 80 (Germany) combination Platinum Diamond ATR, which uses a diamond disk as an internal reflector in the 4000–400 cm^−1^ range. The resolution is 4 cm^−1^, and the refractive index is 2.4^20^.

### Zeta potential analysis

The zeta-potential (calculated as surface charge) experiments of the AgNPs with *P. commune* NRC 2016-3 were performed using the Zetasizer NanoZS Instrument (Malvern, UK). This instrument can determine the zeta potential ranging (mV) between − 200 and 200 mV^24^.

### Treatment fabrics with biosynthesized AgNPs

Biosynthesized AgNPs were applied to several natural fabrics, including silk, cotton, linen, and wool, as shown below. A gram of each fabric was put in 40 mL of the produced AgNPs slurry and heated for 60 min. The fabrics were next rinsed and washed for 15 min at 60^o^C with a solution that includes 2 g/l non-ionic detergents (Triton X-100) and 1.5 g/l Na_2_CO_3_, followed by drying at room temperature^8^.

### Antimicrobial test of the AgNPs treated textiles using optical density assay

The antibacterial activity of textiles (silk, wool, cotton, and linen) treated with biosynthesized AgNPs using *P. commune* NRC 2016-3, following multiple washing cycles, was assessed using the optical density method^22^. Each tube was inoculated with 50 µL of the fresh microbe strains. The test tubes contained 2 × 2 variously treated fabric materials. Untreated fabric samples (controls) were run in parallel under the same circumstances. The percent of suppression was determined as [(A-B)/A] 100, where A and B measure the optical densities of microbial growth at 620 nm in the control and AgNPs-treated fabrics, respectively.

## Results

### Study the antimicrobial activity of the different microorganisms

We used the antimicrobial activity of a well diffusion assay to evaluate the synthesis of AgNPs from extracellular sources for 10 different microbes, the microbial filtrate, and the solution of silver nitrate (control) against the tested microbial isolates. This was carried out using agar well diffusion in Table 2. The obtained results revealed that the AgNPs with *P. commune* NRC 2016-3 displayed the highest antimicrobial activity against the different test microorganisms, and those exhibited mainly inhibiting impacts towards Gram-negative bacteria, exhibiting activity around 26 and 30 mm, Gram-positive bacteria between 30 and 33 mm, candida’s impact was 24 mm, and *A. nige*r’s was 20 mm. Table 3 shows that the MIC of AgNPs evaluated with *P. commune* NRC 2016-3 ranged from 2 to 5 µg/mL for all tested microbes. MBC of the tested AgNPs with *P. commune* NRC 2016-3 was recorded to be 20–30 µg/mL for all tested microorganisms as shown in Table 3.

**Table 2:** Mean zones of growth Inhibition of nanoparticles and reference drugs against test organisms.

Sample	B. subtilus	St. aureus	E. coli	*P*. aeruginosa	C. albicans	A. niger
*B. cereus* 3SME Filtrate	-ve	-ve	-ve	-ve	-ve	-ve
*AgNP with B. cereus* 3SME	21 ± 0.13	23 ± 0.20	24 ± 0.19	27 ± 0.32	30 ± 0.17	16 ± 0.29
*B. velezensis* 3SME	-ve	-ve	-ve	-ve	-ve	-ve
*AgNP with Bacillus velezensis* 3SME	24 ± 0.25	24 ± 0.27	25 ± 0.17	25 ± 0.29	26 ± 0.23	20 ± 0.24
*P. commune* NRC 2016	-ve	-ve	-ve	-ve	-ve	-ve
*AgNP with P. commune* NRC 2016	27 ± 0.27	24 ± 0.29	27 ± 0.34	25 ± 0.27	23 ± 0.31	17± 0.15
*P. commune* NRC 2016-1	-ve	-ve	-ve	-ve	-ve	-ve
*AgNP with P. commune* NRC 2016-1	24 ± 0.25	21 ± 0.22	21 ± 0.23	23 ± 0.17	22± 0.14	16± 0.18
*P. commune* NRC 2016-2	-ve	-ve	-ve	-ve	-ve	-ve
AgNP with P. commune NRC 2016-2	28± 0.16	21± 0.26	20 ± 0.24	21± 0.34	21± 0.19	18± 0.29
P. commune NRC 2016-3	-ve	-ve	-ve	-ve	-ve	-ve

Zones of growth inhibition = diameter of well plus zone of growth inhibition; diameter of well = 9 mm. The mean zone of inhibition was determined from three independent results (n) = 3; nd = not determined, Amp = Ampicillin; Myco = Mycostatin.

**Table 3:** Minimum inhibitory concentration and minimum bactericidal concentration of nanoparticles using well diffusion against test organisms using optical density against test microorganisms.

	B. subtilus	S. aureus	E. coli	*P*. aeruginosa	C. albicans
MIC Inhibition zone µg/mL	≤2	≤ 2	≤ 5	≤ 3	≤5
MBC Inhibition zone µg/mL	≥ 20	≥ 20	≥ 30	≥ 25	≥30

MIC: minimum inhibitory concentration, MBC: minimum bactericidal concentration.

### Biosynthesis and characterization of *P. commune* NRC 2016-3 AgNPs

**Fig. 1:**
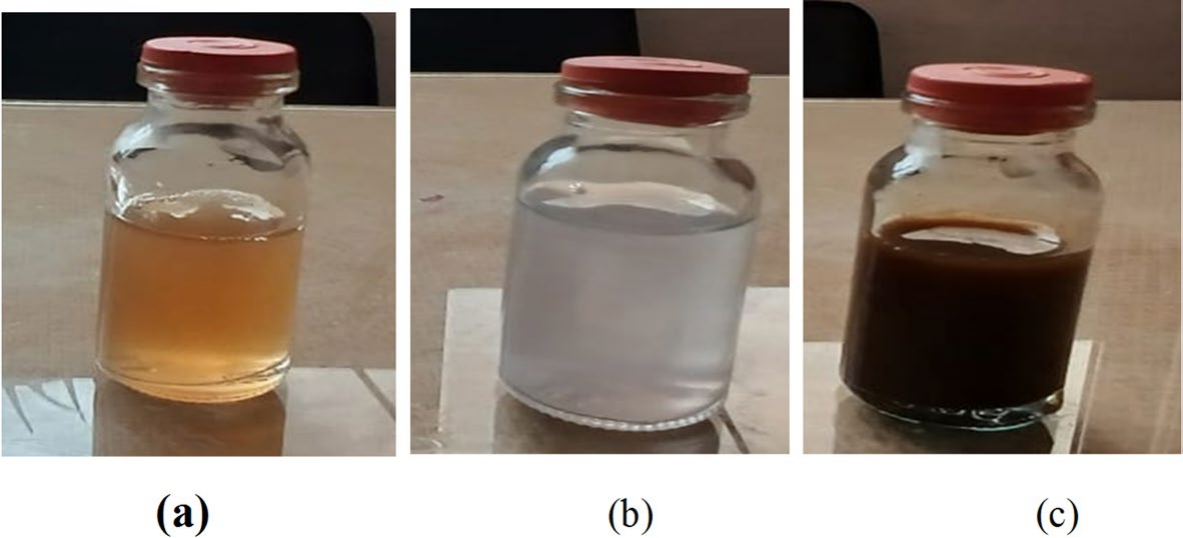
Change in color (**a**) *the P. commune* NRC 2016-3 filtrate, (**b**) after adding AgNO₃ solution to *P. commune* NRC 2016-3 filtrate, and (**c**) a solution containing *P. commune* NRC 2016-3 AgNPs.

**Fig. 2:**
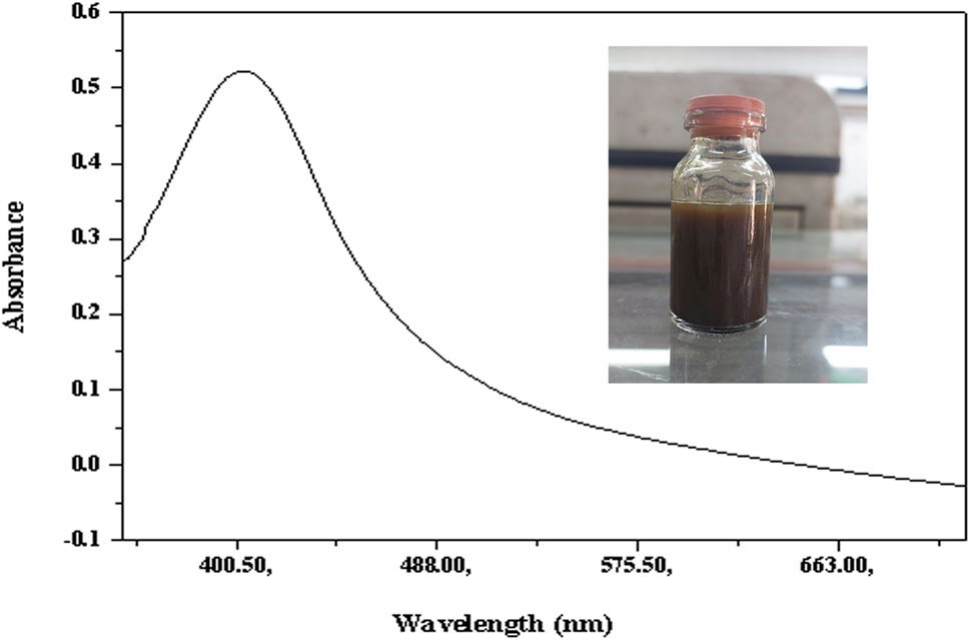
UV–absorbance spectra of biosynthesized silver nanoparticles using *P. commune* NRC 2016-3.

The *P. commune* NRC 2016-3 addition to an aqueous silver nitrate solution formed silver nanoparticles at room temperature. These nanoparticles are yellowish-brown in solution (Fig. 1). The synthesis of AgNPs was further confirmed through UV–vis spectroscopy, utilizing a UV/VIS PC-Spectrophotometer (Shimadzu, UV-2401). UV–vis spectroscopy is a widely recognized and effective method for primarily characterizing synthesized nanoparticles. The UV–vis absorption spectra revealed a broad surface plasmon resonance peak at 420 nm (Fig. 2).

### Transmission electron microscopy (TEM)

**Fig. 3:**
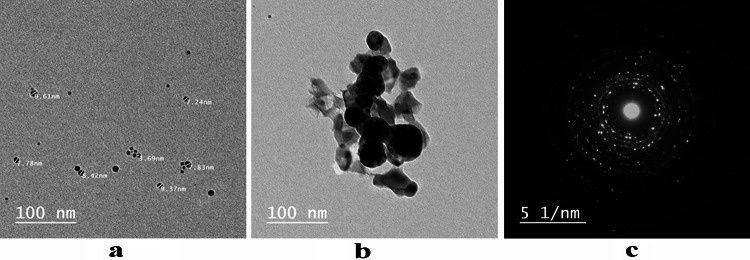
(**a**, **b**) TEM images (**c**) SAED analysis of *P. commune* NRC 2016-3 AgNPs.

The size and morphology of the particles were determined using TEM with the selected area electron diffraction (SAED) analysis approach. Figure 3 (a & b) displayed that the particles are spherical in nature with an average particle size ranging from 3.69 to 9.61 nm. The crystalline structure of the AgNPs was demonstrated through selected area electron diffraction (SAED) analysis, Fig. 2c, which revealed distinct bright concentric circular spots. This confirms the successful biosynthesis of silver nanoparticles.

### Scanning electron microscopy (SEM)

**Fig. 4:**
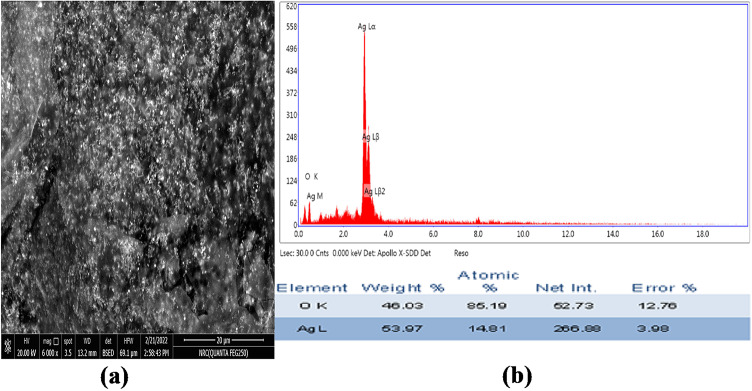
(**a**) SEM, (**b**) EDX analysis of *P. commune* NRC 2016-3 AgNPs.

The surface morphology of biosynthesized AgNPs using *P. commune* NRC 2016 were analyzed by scanning electron microscope (SEM) to explore the surface morphology and topography, and the images are presented in Fig. 4. SEM analysis revealed that AgNPs were polydispersed, predominantly spherical and ranged in size from 3.69 to 9.61 nm. The EDX spectrum indicated the presence of silver, with a weight% of 53.97%, and the elemental composition data is summarized in Fig. 4b.

### X-ray diffraction

**Fig. 5:**
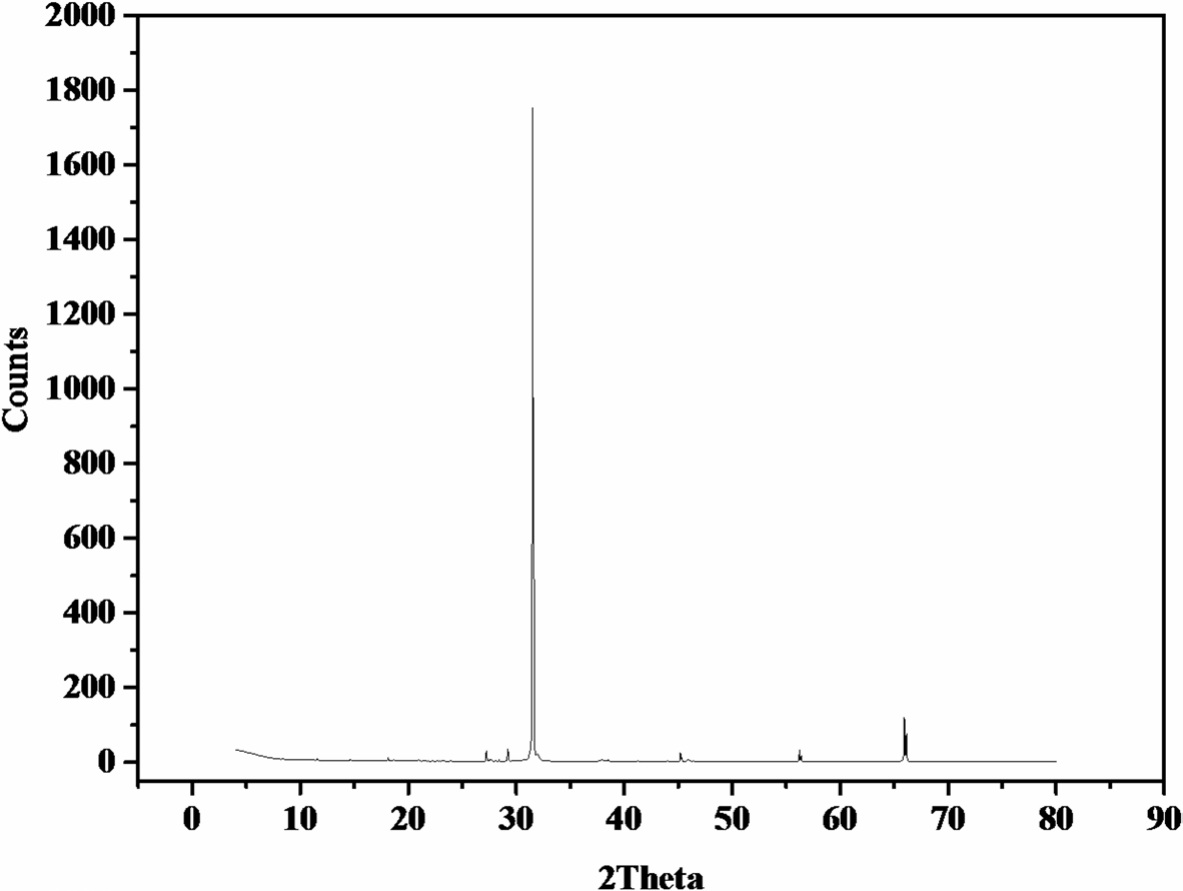
X-ray image of *P. commune* NRC 2016-3 AgNPs.

The crystal structure nature of the synthesized AgNPs by *P. commune* NRC 2016-3 was assessed through XRD, as shown in Fig. 5. The XRD analysis revealed distinct diffraction peaks at 31.5°, 43.6°, 57.1°, and 74.9° indexed to the (111), (200), (220), and (311) planes, as shown in Fig. 5.

### Fourier transforms infrared spectroscopy

**Fig. 6:**
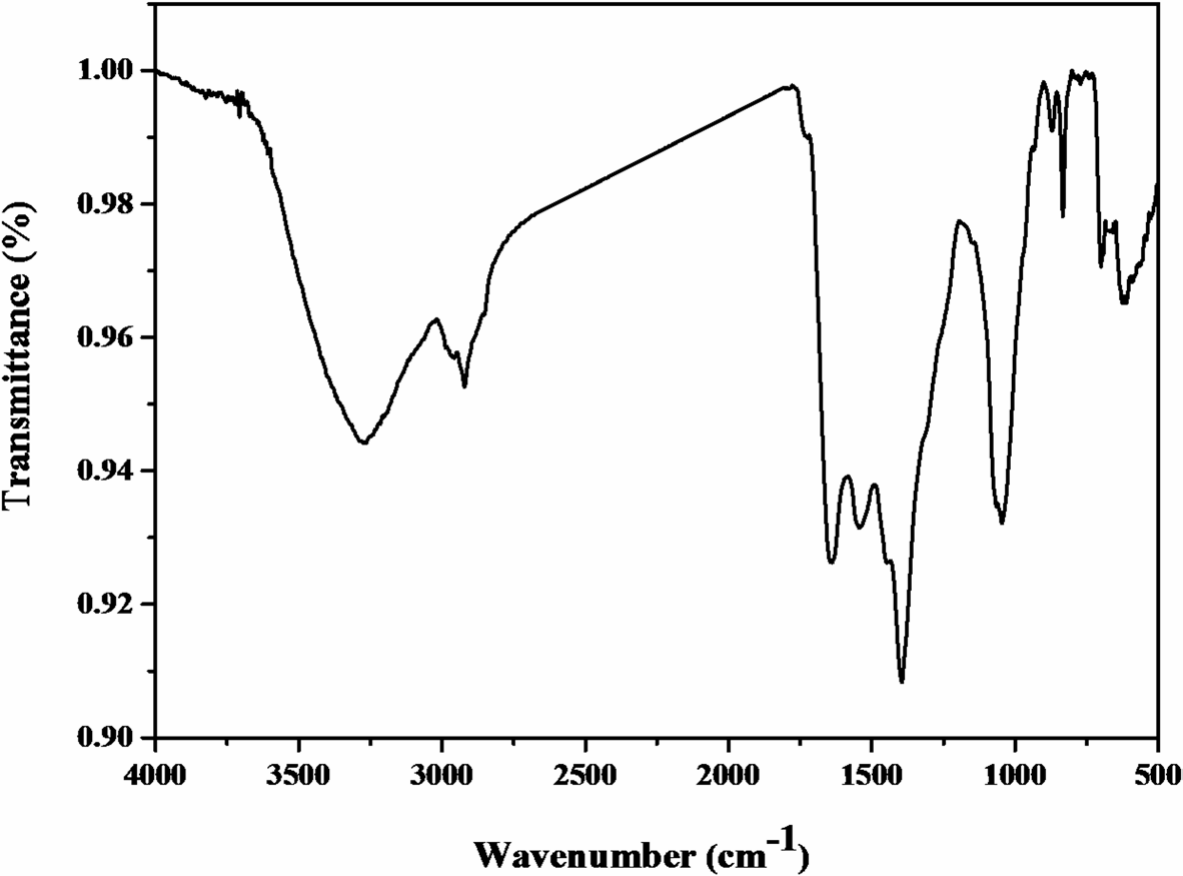
FTIR spectrum of *P. commune* NRC 2016-3 AgNPs.

The FTIR spectrum of AgNPs biosynthesized by *P. commune* NRC 2016-3 as shown in Fig. 6. The FTIR spectrum of silver nanoparticles showed thirteen distinct peaks: 3721.65, 3706.68, 3273.60, 2921.09, 1638.81, 1543.63, 1395.06, 1045.84, 870.64, 832.87, 700.44, 660.43, and 612.07 cm^−1^. The main absorption bands were observed at 2921.09, corresponding to the OH of carboxylic acid, and at 3273.06 cm^−1^, related to the N-H amine group. The peaks at 1359.06 and 1638.81 cm^−1^, correspond to aromatic amines (N–H) of amide A, overlapped by (C–N) of amide I and (C ¼ O) of amide I, respectively. Furthermore, the C-C-O asymmetric stretching band appeared at 1045 cm^−1^.

### Zeta potential analysis

**Fig. 7:**
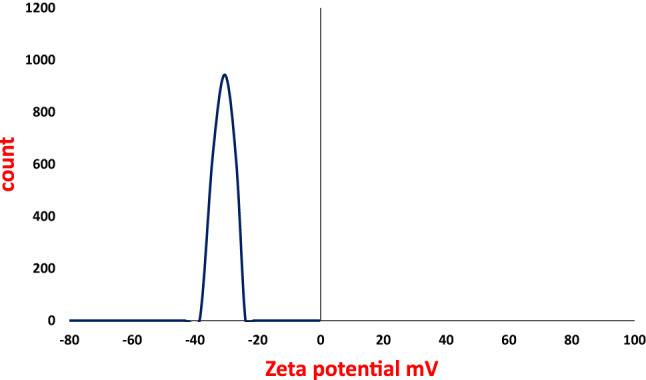
Zeta potential of AgNPs synthesize with *P. commune* NRC 2016-3.

The zeta potential value of AgNPs synthesized with *P. commune* NRC 2016-3 was − 30.5 mV, as shown in Fig. 7.

### Application of AgNPs with *P. commune* NRC 2016-3 for antimicrobial protective textiles

This study investigates the antibacterial and laundering durability of biosynthesized AgNPs using *P. commune* NRC 2016-3 on diverse natural fabrics, including silk, cotton, linen, and wool, to assess their potential for enhancing antimicrobial properties. To assess the durability of AgNPs-treated textiles under repeated washing, the AATCC Test Method 61-2006 was employed. The antibacterial effectiveness of the treated textiles against different pathogenic microbes was evaluated after 1, 5, and 10 laundering cycles, with the corresponding data presented in Table 4. The results showed that the AgNPs-treated textiles exhibited good antibacterial properties against both Gram-positive and Gram-negative bacteria, maintaining their effectiveness even after numerous washing cycles.

**Table 4:** Antimicrobial activities and laundering durability of AgNPs with *P. commune* NRC 2016-3 finished fabrics using optical density assay against microbial test organisms.

Sample		*B. subtilus*	*S. aureus*	*E. coli*	*P*. *aeruginosa*	*C. albicans*
		%				
Silk	1	62.78	67.95	69.24	28.74	61.57
	5	37.51	61.97	47.28	27.71	49.23
	10	29.82	39.31	34.4	14.94	35.87
Linen	1	63.9	30.54	31.39	29.09	74.87
	5	34.69	11.39	22.07	11.78	44.6
	10	10.13	-ve	0.04	8.72	36.55
Wool	1	61.66	52.77	58.71	20.23	82.58
	5	48.82	48.31	54.52	10.29	56.33
	10	47.68	45.02	43.85	3.12	34.05
Cotton	1	35.92	51.67	45.87	45.76	61.86
	5	24.61	45.75	31.02	35.23	59.84
	10	17.71	5.04	28.4	14.42	48.45

## Discussion

In recent years, there has been a growing interest in nanoparticles and their diverse applications. A significant development in this field is nano-biotechnology, which utilizes biological systems such as algae, bacteria, fungi, viruses, and plants for nanoparticle synthesis. Fungi have emerged as effective bio-factories for the synthesis of nanoparticles, offering precise control over particle size and morphology. Silver nanoparticles (AgNPs) synthesized via these methods exhibit potent antimicrobial properties through multiple mechanisms. These include adhesion to microbial cell surfaces, internalization into cells, generation of reactive oxygen species (ROS) and free radicals, and disruption of microbial signaling pathways^26^. The present study aimed to use the Egyptian microbial strain Penicillium commune NRC 2016-3 to biosynthesize silver nanoparticles (AgNPs) and to evaluate their antibacterial and antifungal activities. The successful biosynthesis of AgNPs was initially confirmed by a visible color change of the reaction mixture from yellowish to dark brown upon adding *P. commune* NRC 2016-3 to an aqueous silver nitrate solution. After incubating the reaction mixture overnight in a dark room, the initially colorless solution turned dark brown, indicating that AgNPs had been successfully formed. This color shift is attributed to the excitation of surface plasmon resonance (SPR) characteristic of AgNPs. Furthermore, a wide absorption peak observed at 420 nm using UV–visible spectroscopy confirmed the extracellular synthesis of AgNPs. This peak results from the excitation of surface electrons in the conduction band, a phenomenon associated with the surface plasmon resonance of silver nanoparticles. Previous research indicates that spherical silver nanoparticles typically contribute to absorption bands within the 400–420 nm range in UV–vis spectra. TEM combined with the selected area electron diffraction (SAED) analysis, confirmed the successful biosynthesis of AgNPs with a predominantly spherical shape and an average particle size ranging from 3.69 to 9.61 nm. The crystalline nature of the AgNPs was further demonstrated by the SAED pattern, which displayed distinct bright concentric rings, characteristic of well-defined crystalline structures. Our findings agreed with those of Ingle et al.^27^, who reported the size of AgNPs utilizing TEM of the extracellular *Fusarium acuminatum*, which ranged from 13 nm. SEM analysis indicated that the resulting AgNPs were polydispersed and had apparent spherical morphologies with sizes ranging from 3.69 to 9.61 nm, consistent with TEM analysis.

The analysis of EDX can identify the minerals in the produced nanoparticles, and the various minerals discovered in the produced nanoparticles were attributable to the sample’s pre-treatment before analysis^28^. In the present investigation, the chemical elemental composition and surface atomic distribution of synthesized *P. commune NRC* 2016-3 AgNPs as the silver surface layer were indicated, and those results agreed with the previous study by Khan et al.^4^.

FTIR spectrum confirms the involvement of fungal biomolecules in the synthesis of AgNPs. The spectrum indicated that the biosynthesized AgNPs were capped by the biomolecules secreted by *P. commune* NRC 2016-3, which played a role in reducing Ag⁺^1^ to Ag⁰ and stabilizing the nanoparticles. These findings strongly suggested the presence of a protein covering AgNPs, which could aid in the reduction and stabilization of biosynthesized AgNPs. This observation aligns with previous research conducted by Singh et al.^29^, who synthesized AgNPs. From *Penicillium* sp. Zeta potential estimates an electrostatic charge on the nanoparticle surface that suggests a degree of stability^30,31^. As a current result, the nanoparticle’s zeta potential shows the electrical surface of the particle features, and the large negative zeta potential inhibits particle aggregation, resulting in significant double-layer electrical repulsion. These results are by Liaqat et al.^32^, who reported the AgNPs biosynthesized zeta potential values with *Terminalia* arjuna and *Eucalyptus* camaldulensis were − 20 and − 26 mV, respectively. X-ray diffraction (XRD) analysis confirmed the crystalline nature of the biosynthesized AgNPs by *P. commune* NRC 2016-3. Distinct diffraction peaks were observed at 31.5°, 43.6°, 57.1°, and 74.9°, corresponding to the (111), (200), (220), and (311) planes of face-centered cubic (fcc) silver^4,33^. Minor shifts in peak positions may be attributed to particle size effects or the capping and stabilizing effects of fungal biomolecules during the biosynthesis process.

The antimicrobial activity of a well diffusion assay as a screening methodology was used to evaluate the synthesis of AgNPs from extracellular sources for ten different microbes, the microbial filtrate (negative control), and the silver nitrate solution (control) against the tested microbial isolates. Fungi are better than bacteria and actinomycetes in the significant applications for silver nanoparticles, which lead directly to increased nanoparticle productivity because fungi are more effective than other types of organisms due to their strong affinity for heavy metals^34,35^. The obtained results revealed that the AgNPs with *P. commune* NRC 2016-3 displayed the highest antimicrobial activity against the different test microorganisms. The antimicrobial test exhibited mainly inhibiting impacts towards Gram-ve bacteria, Gram + ve bacteria, candida, and *A. nige*r. This investigation is like a study by Desai et al.^36^, who applied *Penicillium notatum* to synthesize AgNPs and use these as effective antimicrobials against nosocomial infections. Also, Constantin et al.^37^ reported that *Ganoderma lucidum* is commonly used to produce AgNPs that are highly antibacterial and antifungal. In another study by Shelar et al.^38^, they described the extracellular production of AgNPs using the fungus *Fusarium semitectum*, which is effective against *Klebsiella pneumoniae* and *P. aeruginosa*.

Textiles are excellent substrates for cultivating a wide range of microorganisms due to their close contact with the human body and the favorable conditions they provide, such as suitable temperature and humidity. In recent years, growing public concern about hygiene has spurred extensive research into the antimicrobial surface modification of textiles. Among these innovations, metal nanoparticles, particularly silver nanoparticles (AgNPs), have gained considerable attention for their applications across medical, industrial, and environmental fields, thanks to advancements in nanotechnology. This study explored the antimicrobial effectiveness and laundering durability of biosynthesized AgNPs using *P. commune* NRC 2016-3, on diverse natural fabrics. The treated textiles either retained or enhanced their antimicrobial performance after multiple washing cycles, which may be attributed to the disaggregation of AgNPs during laundering. This finding is consistent with Dura’n et al.‘s^39^ findings that AgNPs biosynthesized with *F. oxysporum* may be incorporated into fabric materials to reduce or avoid infection *via S. aureus* bacteria. Similarly, El-Bendary et al.^10^ reported that AgNPs from *B. subtilis*-treated fabric materials had strong antimicrobial effects against several infectious microbes. The successful eco-friendly biosynthesis of small, spherical AgNPs highlights a sustainable alternative to conventional chemical and physical methods that typically require hazardous substances and high energy inputs. These nanoparticles show strong potential for use in antimicrobial textiles and medical applications, such as wound dressings and hospital fabrics, supporting the development of safer, more sustainable healthcare materials.

## Conclusion

The development of green nanotechnology has paved the way for safe, simple, and cost-effective approaches for a wide range of applications. This natural approach uses microbial or plant extracts as more effective agents for stabilizing and reducing the noble metals from their ionic forms into nanoparticles within a stabilized colloidal system. In this investigation, we evaluated the environmentally friendly generation of AgNPs with extracellular filtrate from the fungus *P. commune* NRC 2016-3 strain. The biosynthesized AgNPs showed strong antimicrobial activity against Gram-positive, Gram-negative, and pathogenic fungi. Various techniques, including UV-Vis, TEM, SEM-EDX, XRD, FTIR, and zeta potential, were employed to analyze the fungal biosynthesized AgNPs, which were crystalline in shape, well distributed, with varied shapes and sizes ranging from 3.69 to 9.61 nm, and exhibiting an absorbance peak at 420 nm. Fabrics treated with biosynthesized AgNPs revealed effective antimicrobial activity against harmful microorganisms. The future implications of developing silver nanoparticle synthesis from the *P. commune* NRC 2016-3 strain will allow for manufacturing fabrics that might avoid the growth of microbes and have potential in various industries, especially hospitals.

## Data Availability

The datasets generated during and analyzed during the current study are available including P. commune NRC 2016 strain, Fusarium oxysporum NRC 2017, Bacillus velezensis 3SME, and Bacillus cereus 3SM that – can be deposited in any INSDC member repository at: https://blast.ncbi.nlm.nih.gov/Blast.cgi and the accession numbers to datasets KU752217, MF62208, MW523035, and MW522550 respectively.
